# Ownership and use of mobile phones among health workers, caregivers of sick children and adult patients in Kenya: cross-sectional national survey

**DOI:** 10.1186/1744-8603-9-20

**Published:** 2013-05-14

**Authors:** Dejan Zurovac, Gabriel Otieno, Samuel Kigen, Agneta M Mbithi, Alex Muturi, Robert W Snow, Andrew Nyandigisi

**Affiliations:** 1 Malaria Public Health Cluster, KEMRI-Wellcome Trust-University of Oxford Collaborative Programme, Nairobi, Kenya; 2 Centre for Tropical Medicine, Nuffield Department of Clinical Medicine, University of Oxford, Oxford, UK; 3 Center for Global Health and Development, Boston University School of Public Health, Boston, Massachusetts, USA; 4 Division of Malaria Control, Ministry of Public Health & Sanitation, Nairobi, Kenya; 5 Management for Sciences of Health, Nairobi, Kenya

## Abstract

**Background:**

The rapid growth in mobile phone penetration and use of Short Message Service (SMS) has been seen as a potential solution to improve medical and public health practice in Africa. Several studies have shown effectiveness of SMS interventions to improve health workers’ practices, patients’ adherence to medications and availability of health facility commodities. To inform policy makers about the feasibility of facility-based SMS interventions, the coverage data on mobile phone ownership and SMS use among health workers and patients are needed.

**Methods:**

In 2012, a national, cross-sectional, cluster sample survey was undertaken at 172 public health facilities in Kenya. Outpatient health workers and caregivers of sick children and adult patients were interviewed. The main outcomes were personal ownership of mobile phones and use of SMS among phone owners. The predictors analysis examined factors influencing phone ownership and SMS use.

**Results:**

The analysis included 219 health workers and 1,177 patients’ respondents (767 caregivers and 410 adult patients). All health workers possessed personal mobile phones and 98.6% used SMS. Among patients’ respondents, 61.2% owned phones and 71.4% of phone owners used SMS. The phone ownership and SMS use was similar between caregivers of sick children and adult patients. The respondents who were male, more educated, literate and living in urban area were significantly more likely to own the phone and use SMS. The youngest respondents were less likely to own phones, however when the phones were owned, younger age groups were more likely to use SMS. Respondents living in wealthier areas were more likely to own phones; however when phones are owned no significant association between the poverty and SMS use was observed.

**Conclusions:**

Mobile phone ownership and SMS use is ubiquitous among Kenyan health workers in the public sector. Among patients they serve the coverage in phone ownership and SMS use is lower and disparities exist with respect to gender, age, education, literacy, urbanization and poverty. Some of the disparities on SMS use can be addressed through the modalities of mHealth interventions and enhanced implementation processes while further growth in mobile phone penetration is needed to reduce the ownership gap.

## Background

Mobile health or mHealth is broadly defined as the use of mobile devices such as phones, to support medical and public health practice [1]. mHealth has particularly gained momentum in Africa where rapid growth in mobile phone penetration has been seen as a potential solution to lever human, economic and infrastructure weaknesses of the health system [2,3]. Specifically, the use of the least expensive mobile phone function, text-messaging or technically referred to as SMS for Short Message Service, has been recently deployed in many small scale projects to facilitate disease and outbreak surveillance, supply chain, treatment compliance, quality of care, hospital attendance and public health awareness [4]. Despite a paucity of evaluations to demonstrate the effectiveness and cost-effectiveness of SMS interventions, those interventions targeting patients’ adherence to medications, health workers’ adherence to guidelines and post-treatment attendance have however shown significant outcome improvements during recent randomized trials [5-9] and further studies are either underway or planned [10,11]. Furthermore, several studies applying plausibility designs have shown rather convincing evidence of the impact of SMS reporting on the facility stock-outs for life saving therapies [12,13].

Beside the impact evidence, an important aspect for policy makers planning to adopt SMS interventions is a measure of mobile phone ownership and use among target populations of health workers and their patients at health facilities [14,15]. Kenya is commonly viewed as mHealth hub of East Africa and all reported SMS trials have to-date been undertaken in Kenya [5-9]. However, there have been no published studies reporting coverage data on phone ownership and usage among health workers and patients. We report here recent national data on mobile phone ownership and use among health workers’ and patients’ in Kenya and examine factors influencing ownership and SMS use to help guide the policy implications of mHealth.

## Methods

### Survey design and data collection

A national health facility survey was undertaken between 19^th^ March and 16^th^ April 2012. The survey was part of regular assessments undertaken by the MoH’s Division of Malaria Control to monitor quality of malaria case-management in Kenya. Details of survey methods are presented elsewhere [16,17]. Briefly, the survey was cross-sectional, cluster sample survey undertaken at 172 public health facilities countrywide. National representativeness was assured drawing a stratified random sample of facilities. The facilities from Nairobi and North Eastern provinces were excluded from the sampling frame; the former because of the absence of malaria transmission and the latter due to insecurity in areas afflicted by the war conflict in the neighboring Somalia. A cluster was defined as all encounters between health workers and patients occurring on a survey day.

Data at each facility were collected over a single survey day and included a range of quality-of-care methods. Of relevance for this report, all health workers who saw patients presenting for an outpatient visit were interviewed at the end of the survey day. Similarly, all caregivers of sick children and adult out patients presenting with non-severe febrile disease during the survey day were interviewed at the end of their facility visit. During the interviews all respondents were asked about their demographics, availability of mobile phone networks at facilities and patients’ homes, and patterns of access, ownership and use of mobile phones. Health workers’ willingness to receive text-messages on recommended clinical practices and their preferences for case-management topics were established. Similarly, information on patients’ willingness to receive text-messages about their treatment or treatment of their child was also collected. Finally, the type of mobile phones owned was recorded for all respondents who brought the phone to the health facility on the day of the survey. All health workers, caregivers and adult patients provided written informed consent. Ethical approval for the study was provided by the Kenyatta National Hospital/University of Nairobi-Ethics & Research Committee (KNH-ERC/A/383).

### Data management and statistical analysis

Data entry and management was undertaken using Access (Microsoft, USA). All forms were entered twice by independent data entry clerks and data files were compared for errors using a verification programme and referring to original questionnaires. All analyses were performed using STATA, version 11 (Stata Corp, College Station, Texas).

Descriptive analysis was undertaken for health workers, caregivers of sick children and adult patients as well as on combined set of respondents comprising both caregivers and adult patients. For the purposes of analysis adult patients were defined as individuals 15 years and older, the age above which outpatients are likely to visit health facilities unaccompanied, have mobile phones and would be potential direct recipients of SMS interventions improving adherence to medicines. The primary study outcomes were proportions of respondents 1) owning a personal mobile phone and 2) using SMS among phone owners. Access to mobile phone was defined as either personally owned phone or respondent’s access to the mobile phone owned by another member of the same household. The SMS use was defined as routine sending and receiving of unformatted text-messages. In addition to text-messaging, the use of mobile phones was assessed for voice communication (defined as making and receiving calls), mobile money transfers, internet browsing, and e-mail communication. For respondents who possessed their personal phones at the time of the interview, their mobile devices were classified as basic (voice and text-messaging only), medium (limited data transfer possible but not on “smartphone” operating systems) and “smartphones” (devices with Android, Symbian, iOS and Blackburry operating systems).

To explore factors influencing personal ownership of mobile phones and SMS use among phone owners the following factors were examined using cluster adjusted logistic regression: gender, age, education, urbanization, literacy and poverty index. Health workers universally owned phones and used SMS and were therefore not included in the predictors analysis. Since phone ownership and use of SMS was similar between caregivers and adult patients the predictors’ analysis combined both categories of respondents. Education levels were defined as the highest level of education reached. Literacy was defined as ability to read tested by interviewers. For respondents’ urbanization and poverty index status, the proxy measures based on health facility locations were used. The respondents were classified as living in either urban or rural areas based on the urban versus rural census delineations [18]. The poverty index, defined as the percentage of population falling below the poverty line [19], was used to classify respondents into three categories (poverty below 30%, 30-60%, and 60% and above). For both outcomes the odds ratio (OR), 95% confidence interval and *P*-values were first estimated for each factor in a series of univariate models. All factors with *P*-value for association <0.15 were then entered into multivariate model. Hypothesis testing and confidence interval estimations were done with an alpha level of 0.05. *P*-values between 0.05 and 0.10 were considered to be of borderline statistical significance. For descriptive analysis differences in proportions between caregivers and adults patients were tested using cluster adjusted chi-square test.

## Results

### Sample description

The survey included 1,291 febrile patients seen by 222 health workers at 172 health facilities. The majority of facilities were government owned (89.0%), dispensaries (68.6%) and located in rural areas (88.4%). Of 222 health workers who saw patients on survey days, data were available for 219 health workers who were included in the final analysis. The median age of health workers was 35 years [IQR: 29–44] and the majority were female (54.8%) and nurses by profession (62.1%). Of 1,291 patients, 114 (8.8%) were excluded from the analysis because of incomplete data sets. The final analysis therefore included 1,177 interviewed respondents (767 caregivers and 410 adult patients). The median age of caregivers was 28 years [IQR: 23–34) and the majority were female (90.4%), mothers of sick children (81.7%), literate (84.7%) and with partial or completed primary education (61.6%). With respect to adult patients, their median age was 35 years [IQR: 25–51] and the majority were also female (66.6%), literate (76.3%) and with primary education (49.8%). Finally, the majority (55.8%) of all respondents presented to health facilities in areas with over 30% of population living below the poverty line.

### Ownership and use of mobile phones among health workers

Of 219 interviewed health workers, all possessed personal mobile phones and nearly all (98.6%) used SMS (Table 1). The majority (92.2%) worked at facilities with mobile phone network. In addition, nearly all health workers used mobile phones for money transfers (99.5%), approximately half for internet browsing (50.2%) and e-mail communication (47.0%). Of 181 health workers whose phone model could be determined, the majority (56.9%) had medium level phones, 33.7% had basic ones while only 9.4% possessed “smartphones”. The majority (204; 93.2%) of health workers responded that they would like to receive text-message reminders on recommended clinical practices, of which the most commonly reported case-management topics were malaria (89.7%), HIV (44.1%), tuberculosis (36.3%), pneumonia (29.9%), diarrhea (28.4%), typhoid (18.6%), diabetes (18.6%), hypertension (17.7%), upper respiratory tract infections (13.7%) and skin infections (13.2%). In order of preference, malaria was the first choice responded by 73.0% of health workers.

**Table 1:** Mobile phone access, ownership and use among outpatient health workers, caregivers of sick children and adult patients

	**Health workers**	**Caregivers of sick children and adult patients**			
		**Caregivers**	**Adult patients**	*p*-value*	**Both respondents**
**Network coverage, phone access and ownership**	**N = 219**	**N = 767**	**N = 410**		**N = 1,177**
Mobile network coverage	202 (92.2%)	703 (91.7%)	381 (92.9%)	0.448	1,084 (92.1%)
Has access to mobile phone	219 (100.0%)	663 (86.4%)	348 (84.9%)	0.594	1,011 (85.9%)
Has personal mobile phone	219 (100.0%)	464 (60.5%)	256 (62.4%)	0.562	720 (61.2%)
**Use of mobile phones**	**N = 219**	**N = 457**	**N = 255**		**N = 712**†
Voice	219 (100.0%)	454 (99.3%)	254 (99.6%)	0.655	708 (99.4%)
SMS	216 (98.6%)	335 (73.3%)	173 (67.8%)	0.118	508 (71.4%)
Mobile money transfers	218 (99.5%)	386 (84.5%)	217 (85.1%)	0.822	603 (84.7%)
Internet browsing	110 (50.2%)	20 (4.4%)	20 (7.8%)	0.055	40 (5.6)
E-mail	103 (47.0%)	19 (4.2%)	15 (5.9%)	0.339	34 (4.8%)

† Analysis restricted to respondents who had personal mobile phones (8 observations excluded due to missing “use of phone” variables).

* Cluster adjusted chi-square test for difference in proportions between caregivers and adult patients.

### Ownership and use of mobile phones among caregivers and adult patients

Of 1,177 respondents, the large majority (92.1%) reported living in households with a mobile phone network, 85.9% had access to mobile phones within the household while 61.2% owned a personal mobile phone without significant difference between caregivers and adult patients (60.5% vs 62.4%; p = 0.562) (Table 1). Among phone owners, nearly all (99.4%) respondents used the voice function of mobile phones while the use of text-messaging, although high, was less common (71.4%). There was higher use of text-messaging among caregivers (73.3%) compared to adult patients (67.8%) however without statistically significant difference (p = 0.118). The use of mobile phones for money transfers was common among all respondents (84.7%) while the use of phones for internet browsing (5.6%) and e-mail (4.8%) was rare. Of 423 respondents who brought the phone to the facility and whose model of the phone could be established, the large majority (81.6%) had basic phones, 16.8% had medium level phones while “smartphones” were very rare (1.7%). Finally, nearly all (93.8%) respondents expressed willingness to receive text-messages about their treatment or treatment of their child.

### Predictors of mobile phone ownership and SMS use

We examined the effects of six factors on the respondents’ ownership of mobile phones and use of SMS in data set combining interviews of caregivers and adult patients. In the univariate analysis nearly all factors met our entrance criteria for multivariate model (*P* < 0.15). The only factor not meeting this criterion was the lack of an association between the poverty index and SMS use. Table 2 presents multivariate results for both outcomes after adjusting for covariates. Male respondents were significantly more likely to own mobile phones (OR = 1.75; 95% CI: 1.21-2.51) and use SMS (OR = 1.63; 95% CI: 0.97-2.74). Higher education was significantly associated with both outcomes. For example, compared to respondents without any education those who reached secondary school were more likely to have mobile phones (OR = 3.20; 95% CI = 1.54-6.62) and use SMS (OR = 7.94; 95% CI = 2.42-26.10). Respondents from urban areas were more likely to possess mobile phones compared to those in rural areas (OR = 1.51: 95% CI = 1.03-2.23) as well as literate respondents compared those unable to read (OR = 3.74; 95%: 2.46-5.69). Similarly, urban (OR = 2.00; 95%: 1.19-3.37) and literate respondents (OR = 4.27; 95%: 1.92-9.49) were more likely to use SMS. Interestingly, respondents living in areas with poverty index lower than 30% were significantly more likely to own personal phones compared to respondents living in areas with the index greater than 60% (OR = 2.13; 95% CI: 1.04-4.36), however comparison of the same respondents’ categories has not shown significant association for SMS use (OR = 1.20; 95% CI: 0.53-2.70). With respect to respondents’ age, the youngest category (15–19 years) was less likely to own the phones, however when the phones are owned by respondents, younger age groups were significantly more likely to use SMS (Table 2).

**Table 2:** Factors influencing ownership of mobile phones and use of SMS among caregivers and adult patients: results of multivariate analysis

**Factor**	**Mobile phone ownership (N = 1,167)**				**Use of SMS (N = 712)**			
	**N**	**n (%)**	**OR (95% CI)**	**p-value**	**N**	**n (%)**	**OR (95% CI)**	**p-value**
**Gender**								
Male	211	151 (71.6)	1.75 (1.21-2.51)	0.003	150	117 (78.0)	1.63 (0.97-2.74)	0.065
Female	956	565 (59.1)	**1.0 (Ref.)**		562	391 (69.6)	**1.0 (Ref.)**	
**Education**								
Higher	46	44 (95.7)	23.38 (4.92-111.14)	<0.001	44	42 (95.5)	33.04 (6.14-177.73)	<0.001
Secondary	295	220 (74.6)	3.20 (1.54-6.62)	0.002	219	189 (86.3)	7.94 (2.42-26.10)	0.001
Primary	675	410 (60.7)	1.78 (0.94-3.37)	0.077	408	270 (66.2)	2.78 (0.89-8.74)	0.079
No formal education	151	42 (27.8)	**1.0 (Ref.)**		41	7 (17.1)	**1.0 (Ref.)**	
**Age**								
15-19 years	83	24 (28.9)	0.17 (0.07-0.38)	<0.001	24	19 (79.2)	3.82 (1.01-14.50)	0.049
20-29 years	506	313 (61.9)	0.77 (0.44-1.33)	0.311	311	248 (79.7)	3.14 (1.66-5.94)	0.001
30-39 years	300	208 (69.3)	1.07 (0.59-1.94)	0.832	206	148 (71.8)	1.91 (0.96-3.78)	0.064
40-49 years	129	94 (72.9)	1.59 (0.83-3.05)	0.159	94	57 (60.6)	1.20 (0.55-2.58)	0.647
50+ years	149	77 (51.7)	**1.0 (Ref.)**		77	36 (46.8)	**1.0 (Ref.)**	
**Literacy**								
Able to read	916	641 (70.0)	3.74 (2.46-5.69)	<0.001	640	489 (76.4)	4.27 (1.92-9.49)	<0.001
Unable to read	251	75 (29.9)	**1.0 (Ref.)**		72	19 (26.4)	**1.0 (Ref.)**	
**Urbanization**								
Urban area	148	107 (72.3)	1.51 (1.03-2.23)	0.037	106	89 (84.0)	2.00 (1.19-3.37)	0.009
Rural area	1,019	609 (59.8)	**1.0 (Ref.)**		606	419 (69.1)	**1.0 (Ref.)**	
Poverty index*								
<30% of population	100	75 (75.0)	2.13 (1.04-4.36)	0.038	75	56 (74.7)	1.31 (0.57-3.00)	0.519
30-60% of population	552	349 (63.2)	1.37 (0.97-1.94)	0.074	346	245 (70.8)	0.92 (0.59-1.46)	0.733
>60% of population	515	292 (56.7)	**1.0 (Ref.)**		291	207 (71.1)	**1.0 (Ref.)**	

* The results of univariate analysis on the effects of poverty index on SMS use are presented but not included in the multivariate model.

## Discussion

Our health facility survey undertaken in 2012 in Kenya revealed universal mobile phone ownership and SMS use among health workers, lower phone ownership (61%) and SMS use (71%) among caregivers of sick children and adult patients, and significant disparities among patients in relation to gender, age, education, literacy, urbanization and poverty status.

With respect to health workers and mobile phone ownership, our findings demonstrate high readiness of Kenyan public health sector to support large scale implementations of SMS based interventions without the need for supply of mobile devices. Moreover, widespread use of text-messaging will be undoubtedly beneficial to facilitate training requirements for implementation of interventions requiring SMS communication with health workers [7,12]. Yet, we acknowledge that the readiness of Kenyan health workers to support more complex, data-based technology interventions requiring newer generation of mobile devices, such as “smartphones” [20], is still at an early stage. Presently, and prior to demonstrating their effectiveness, the implementation of such interventions would still require large scale procurement and distribution of mobile devices which should be accompanied by on-going interventions to ensure appropriate and sustained use of such devices [20,21].

Patients presenting to public health facilities had a lower phone ownership than health workers. Despite a high access (86%) to mobile phones within households, nearly 40% of patients do not have personal phones. For SMS interventions targeting individual patients, personal ownership of phones is highly desirable to ensure privacy of transmitted information and maximize exposure to SMS interventions which is likely to be lower for shared phones. More positively, 61% of patients owning a phone in our survey is substantially higher compared to 44% of phone owners reported during the national household survey in 2009 [22]. Comparisons between self-selected populations presenting to facilities and healthy respondents at households are prone to limitations, however it is reasonable to assume that further growth in mobile phone penetration within general population will be reflected in increased phone ownership among patients – the population category of particular interest for policy makers implementing facility-based interventions. We also observed no significant difference in phone ownership between caregivers of sick children and adult patients; the finding likely influenced by the fact that the majority of population presenting to public facilities in Kenya are female either seeking care for themselves or for their children.

Patients’ use of SMS among phone owners was also not universal. The finding that nearly 30% of respondents are not SMS users highlights the fact that the ability to communicate via SMS should not be assumed. The future text-message interventions targeting individual patients should devote time not only to the training on specifics of SMS interventions, but also to the basics of SMS communication. Major challenges in this process should not be however expected given that the large majority of patients in Kenya do use mobile phones for money transfers using formatted SMS modules.

Our predictors analysis provides additional insights into the patterns of phone ownership and SMS use. Mobile phone owners are more likely to be male, more educated, literate, living in urban areas and in areas of better economic status. With respect to malaria, these trends are unfortunately in contrast with control needs where populations of high malaria risk are children and pregnant women of low socioeconomic status living in rural areas. The same pattern of association was found between these factors and SMS use with an exception to the effect of the poverty level. While respondents living in wealthier areas are more likely to own a phone, no association was however observed between the poverty level and SMS use. The latter suggests that the current low cost of SMS does not present a barrier for SMS use even among the poorest populations while the poverty still remains an important impediment for acquisition of even basic mobile device. With respect to age, the respondents in the youngest age category (15–19 years) are less likely to own phones however when they do have the phones they are more likely to use SMS compared to older respondents. The disparities in SMS use are however mHealth challenges that can be addressed through the careful training of phone owners as well as through the various modalities of mHealth interventions. For example, SMS non-users, and in particular illiterate ones, could be offered a voice communication option, a mobile phone function universally used by all phone owners and indeed a suggested option in recent studies in India [23,24].

An important consideration for success of SMS interventions is health workers’ and patients willingness to receive text messages. We found this to be very high for both categories of potential recipients with 93% of health workers willing to receive SMS reminders on recommended clinical practices and 94% of patients willing to receive text messages on their treatment or the treatment of their child. Our 2012 results concur with 96% of willingness results reported among patients in 2007 in South Africa [25] and suggest that, at least in Kenyan context, SMS interventions are still innovative and exciting. Moreover, the finding that 90% of health workers selected malaria for SMS case-management topic and this was furthermore the topic of the first choice preference for 73% of health workers, suggests that malaria is a disease of the major interest for SMS based interventions in Kenya.

Finally, several limitations of our study should be mentioned. First, exclusion of urban facilities in non-malarious areas in the capital may have underestimated ownership and use of mobile phones among the patients. We believe however that this effect is likely to be minor given the proportionally small number of public facilities in Nairobi compared to other areas and the likelihood of a cancelled bias through the non-inclusion of rural facilities in economically impoverished and security affected North Eastern province. Second, due to the primary objective of the survey to evaluate malaria related care, the inclusion of only febrile patients may have biased inference of results more towards malaria patients. Given that fever is highly common and malaria non-specific presentation, we however also believe that our findings reflect well the general pattern of outpatients presenting to Kenyan facilities. Third, in the absence of assessment of individual patients’ socioeconomic status the use of proxy measures for determination of urbanization and poverty status may have introduced some misclassification. Finally, courtesy bias in responses cannot be ruled out for assessment of willingness to receive SMS interventions.

## Conclusions

Our 2012 findings at health facilities in Kenya demonstrate optimal conditions for SMS based interventions targeting health workers in public sector. The patients they serve have however lower ownership of mobile phones, use SMS less often and disparities exist with respect to gender, age, education, literacy, urbanization and poverty. Some of the disparities on SMS use can be addressed through the modalities of mHealth interventions and enhanced implementation processes while the further growth in mobile phone penetration is needed to reduce the ownership gap.

## Competing interests

All authors declared no competing interest.

## Authors’ contributions

DZ, SK, AMM, AM, RWS and AN contributed to study design, training of field workers and supervision of the field work. GO and DZ analyzed the data. DZ produced the first draft of the manuscript. All authors critically reviewed the paper and approved the final version.

## Authors’ information

DZ is MD, PhD and an epidemiologist within KEMRI-Wellcome Trust-University of Oxford Collaborative Programme in Nairobi. GO is B.Ed Sc, MSc and a statistician within KEMRI-Wellcome Trust-University of Oxford Collaborative Programme in Nairobi. SK is Senior Nursing Officer and Programme Officer within the MoPH’s Division of Malaria Control in Nairobi. AMM is MD, MSc and an epidemiologist within the MoPH’s Division of Malaria Control in Nairobi. AM is BPharm and Senior Technical Advisor at Management for Sciences of Health in Nairobi. RWS is FMedSci, PhD and the Head of Malaria Public Health Cluster within KEMRI-Wellcome Trust-University of Oxford Collaborative Programme in Nairobi. AN is BPharm and the Head of case-management unit within the MoPH’s Division of Malaria Control in Nairobi.
